# Experimental Investigation on the Gas Phase Behaviour and Inhibition for Hydrates with CO_2_-Rich Gas in an Oil–Water System

**DOI:** 10.3390/ma19091795

**Published:** 2026-04-28

**Authors:** Peifen Yao, Xingya Ni, Qiaosheng Zhang, Xiaoming Luo

**Affiliations:** 1 College of Pipeline Engineering, Xi’an Shiyou University, Xi’an 710065, China; 2 College of Pipeline and Civil Engineering, China University of Petroleum (East China), Qingdao 266580, China; 3 Changqing Engineering Design Co., Ltd., China Petroleum and Natural Gas Corporation, Xi’an 710018, China

**Keywords:** high-CO_2_ gas, oil–water hydrate, gas phase state, hydrate inhibitor

## Abstract

**Highlights:**

- Elucidated the effects of P&T-induced gas state on hydrate nucleation and growth.
- Revealed the mechanisms of CO_2_ on kinetics and morphological evolution.
- Clarified the hydrate inhibition mechanisms of methanol and MEG in O-W systems.
- Analyzed the hydrate dissociation and evolution in methanol-inhibited systems.

**Abstract:**

During deepwater oil and gas production and shut-in operations, the high-pressure and low-temperature environment readily induces hydrate formation of CO_2_-rich associated gas in oil–water systems, thereby posing serious flow assurance risks. This study systematically investigated the nucleation, growth, and morphological evolution of hydrates in oil–water systems under different gas-phase states using fully visualized high-pressure apparatus, along with the effects of temperature, pressure, CO_2_ concentration, and inhibitors on hydrate formation behavior. The results showed that gas phase transition significantly altered the hydrate induction time, gas consumption, and growth time. However, once the gas was liquefied, mass transfer became hindered, and the growth process exhibited pronounced dynamic fluctuations. Phase transitions caused by variations in CO_2_ concentration also exerted a significant influence on hydrate growth, among which the terminal subcooling had the most pronounced effect on the integrated growth index. Compared with monoethylene glycol (MEG), methanol lowered the peak value during the rapid hydrate formation stage, markedly reduced the hydrate growth rate, and led to a prolonged period during which the pressure remained above its initial value. These findings revealed the hydrate formation characteristics in oil–water systems and mechanism of thermodynamic inhibitors, providing a theoretical basis for ensuring flow safety in CO_2_-rich oil and gas wellbores and pipelines.

## 1. Introduction

During the development and transportation of deepwater oil and gas resources, production shutdowns may occur as a result of extreme weather events such as typhoons. Under such conditions, subsea production wells remain in a high-pressure and low-temperature environment for extended periods, which readily promotes hydrate formation and pipeline blockage, disrupts the normal transport of oil and gas resources, and, without timely intervention, may damage the pipeline, trigger safety issues, and cause substantial economic losses [1]. Therefore, the effective prevention of hydrate formation, agglomeration, and blockage is of great strategic importance to the oil and gas industry [2]. At present, with the continued expansion of offshore oil and gas development into deeper and more remote waters, the industry is increasingly confronted with the challenge that CO_2_-rich associated gas readily forms hydrates [3]. Sloan et al. [4,5] indicated that the hydrate issue gradually evolved from an early strategy of “complete avoidance” into a flow assurance problem dominated by “risk management,” highlighting the engineering urgency of investigating hydrate behavior under complex operating conditions.

The progression from hydrate formation to pipeline blockage can generally be divided into two stages: the first is hydrate nucleation and growth, in which hydrate cages begin to form once the environmental conditions reach the hydrate phase-equilibrium temperature and pressure, guest molecules are incorporated into these cages, and hydrate nuclei gradually develop; the second is hydrate agglomeration, during which the formed hydrate nuclei attract one another, causing hydrate particles in the flowing fluid to increase in size and eventually deposit in the pipeline after agglomeration reaches a certain extent [6]. Studies by Chen et al. [7] and Akhfash et al. [8] on the mechanism of pipeline blockage showed that the blockage risk depended not only on whether hydrates were formed, but more importantly on the spatial distribution of hydrate particles, their agglomeration patterns, and the rheological evolution of the continuous phase. Therefore, relying solely on the phase-equilibrium boundary is insufficient to reveal the blockage mechanism in actual pipelines, and comprehensive analysis must also incorporate heat transfer, mass transfer, and multiphase flow characteristics.

In actual offshore oil and gas development, the fluid transported in pipelines is usually not a simple gas–water two-phase system, but more commonly a multiphase system in which oil, gas, and water coexist [9]. The presence of the oil phase can significantly alter the location of hydrate formation, the mode of interfacial growth, and the interparticle forces, causing the hydrate formation process to exhibit more complex kinetic characteristics than those in pure water systems [10]. A review of oil-dominated systems indicated that the oil phase not only affects water-droplet dispersion and gas–liquid mass-transfer pathways, but also modifies the agglomeration behavior of hydrate particles through emulsion stability, interfacial film structure, and naturally occurring surface-active components [11]. Guo et al. [12] on hydrate growth at the oil–water interface further showed that hydrates may preferentially form a shell structure at the oil–water interface, and this shell in turn restricts subsequent mass transfer and alters crystal morphology [13]. These findings suggest that hydrate growth in oil–water systems is no longer a simple bulk crystallization process, but instead displayed pronounced interfacial control and multiphase coupling characteristics [14,15,16].

In addition to phase-state structure, gas composition is also a key factor influencing hydrate formation behavior. Because CO_2_ has a strong hydrate-forming tendency and relatively high solubility in water, it usually exerts a significant influence on the hydrate formation boundary and kinetic process in mixed-gas systems. Early summaries of hydrate thermodynamics and formation behavior have shown that differences among guest molecules in solubility, molecular size, and hydrate stability can directly affect the nucleation and growth characteristics of hydrates [17]. In recent years, studies on CO_2_ hydrates and their inhibition have further demonstrated that, at high CO_2_ concentrations, the driving force for hydrate formation, phase-transition behavior, and interfacial mass-transfer mode of the system may all change markedly [18]. In particular, under higher pressures, once CO_2_ liquefied, its partitioning between the oil phase and the aqueous phase becomes more complex, giving rise to hydrate formation pathways that differ from those in conventional gaseous systems [19].

At present, the engineering strategies for hydrate prevention and control mainly include heating, depressurization, dehydration, and the injection of chemical inhibitors. Among these methods, chemical injection remains one of the most commonly used approaches for hydrate prevention and control in subsea pipelines and wellbores because of its operational flexibility and strong adaptability [20]. Existing studies generally classify chemical inhibitors into three categories: thermodynamic inhibitors, kinetic inhibitors, and anti-agglomerants [21]. Among them, methanol and monoethylene glycol (MEG) are the most representative thermodynamic inhibitors, and they can delay hydrate formation by reducing water activity and altering hydrate phase-equilibrium conditions. Therefore, they are the most widely used in industrial practice [22]. Kelland’s summary of the development history of low-dosage hydrate inhibitors [6], together with many subsequent reviews, has shown that although thermodynamic inhibitors were mature and reliable, they still suffered from drawbacks such as high dosage, high recovery cost, and considerable environmental burden. Therefore, clarifying their scope of action and inhibition mechanism in complex multiphase systems remains of great importance [23].

For methanol and MEG, two commonly used thermodynamic inhibitors, existing phase-equilibrium studies have confirmed that both can effectively increase the dissociation pressure of CO_2_ hydrates or lower their formation temperature range, although their inhibition performance and applicability were not entirely the same [24]. The phase-equilibrium study by Dastanian et al. [25] on CO_2_ hydrates in methanol/MEG systems showed that, at comparable mass concentrations, methanol generally exhibited stronger thermodynamic inhibition; related experimental measurements also confirmed that, in saline systems, methanol produced an overall greater shift in the equilibrium conditions of CO_2_ hydrates than MEG. In addition, studies on the thermodynamic behavior of aqueous MEG solutions have shown that, beyond affecting the equilibrium boundary, MEG also significantly altered the liquid-phase properties and phase-partitioning behavior of the system, implying that in oil–water multiphase environments, the effects of these two inhibitors on hydrate nucleation, growth, and dissociation may be reflected not only in whether hydrates form, but also in the formation rate, particle morphology, and dissociation pattern [26].

It should be noted that although substantial progress has been made in studies on hydrate formation and inhibition, most existing work still focuses on pure gas–water systems, water-rich gas-dominated systems, or macroscopic blockage risk assessment [21]. For CO_2_-rich oil–water systems, especially under conditions where the gas phase state may shift from a gaseous state to a gas–liquid coexistence state or even a liquid state, systematic studies on hydrate nucleation, growth, interfacial morphological evolution, and dynamic pressure response remain lacking. In particular, when liquid CO_2_ coexisted with the oil phase, factors such as dissolution–precipitation, hindered interphase mass transfer, changes in interfacial area, and expansion of the mixed-phase space may act simultaneously, causing hydrate formation behavior to deviate markedly from conventional understanding. Meanwhile, the differences in inhibition performance between methanol and MEG in such complex systems, as well as the mechanisms by which they affect dissociation morphology, still lack support from visualized experimental evidence.

This study systematically investigated the hydrate formation characteristics in oil–water systems and the mechanisms of inhibitor action by varying temperature, pressure, and CO_2_ concentration, and by combining the induction time, gas consumption, growth time, and morphological evolution of hydrates under different gas phase states, it clarifies the differences in hydrate formation mechanisms under different phase-state conditions and identifies the effects of gas phase transition, driving force, and subcooling on hydrate formation. The influence of CO_2_ concentration on the hydrate formation mechanism is further analyzed, and the deviation in hydrate formation volume is interpreted on the basis of hydrate growth morphology. The growth mechanisms and kinetic characteristics of hydrates under the effects of different inhibitors are obtained, and the differences and features of typical thermodynamic inhibitors are discussed, thereby providing more effective strategies and a theoretical basis for hydrate blockage prevention and control in oil and gas field development.

## 2. Experiments

### 2.1. Experimental Setup

**Figure 1:**
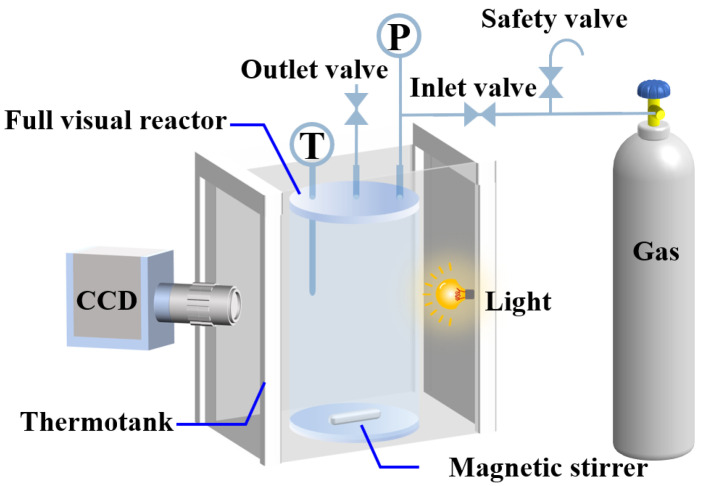
Hydrate formation experimental system and apparatus.

The experiments in this study were conducted using a fully visualized high-pressure hydrate experimental system manufactured by Jiangsu Tuochuang Scientific Research Instrument Co., Ltd. (Nantong, China), and a schematic diagram of the apparatus is shown in Figure 1. The core unit of the system was a fully visualized sapphire reactor with an inner diameter of 40 mm and a height of 210 mm. The reactor lid was equipped with a temperature sensor with an accuracy of 0.1 °C, a pressure sensor with an accuracy of 0.25%, and gas inlet and vent lines. A magnetic stir bar was placed at the bottom of the reactor, and agitation was provided magnetically, with a maximum stirring speed of 1000 r/min. The gas inlet line was fitted with a safety valve, and pressure control up to 60 MPa was achieved through a gas pressurization unit. Experimental temperature control was provided by a programmable constant-temperature chamber, which allowed temperature settings from −20 to 100 °C. An LED light source was installed inside the chamber to ensure sufficient illumination for hydrate observation. The entire experimental process was recorded by a high-definition CCD camera, and data acquisition was precisely managed by a computer-based control system.

### 2.2. Materials

**Figure 2:**
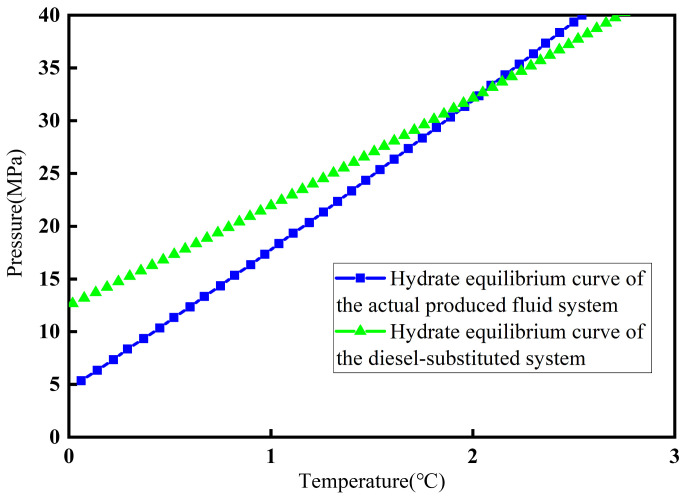
Hydrate phase equilibrium curve of different systems.

Analysis of the actual field-produced fluid showed that it consisted of gas and oil phases. Because the composition of the actual oil phase was highly complex and unfavorable for direct visual observation of hydrate growth behavior, diesel was used as a model oil phase to replace the original oil phase in the produced fluid system. Figure 2 presents the hydrate phase equilibrium curves predicted by CSMGem (version 1.10) for the actual produced fluid system and the diesel-substituted system. The blue curve represents the predicted hydrate phase equilibrium curve corresponding to the actual produced fluid system from the oilfield. The green curve represents the predicted hydrate phase equilibrium curve of the system after replacing the original oil phase with diesel.

Comparison of the hydrate phase-equilibrium curves (Figure 2) shows that the hydrate formation curves of the two systems were similar, indicating that diesel can be used in the experiments. The experimental gas was a custom-prepared gas mixture consistent with the actual field composition, and its composition was listed in Table 1. Accordingly, the experimental materials used in this study included the custom gas mixture, 0# diesel purchased from Shandong Hongda Biotechnology Co., Ltd. (Linyi, China), and a 3.5 wt% NaCl aqueous solution. The deionized water used in the experiments was prepared in the laboratory and had a resistivity of 18.25 MΩ·cm. In the experimental system, the gas-to-oil ratio was 4:3, and the oil-to-water ratio was 1:1.

### 2.3. Experimental Procedure

**Figure 3:**
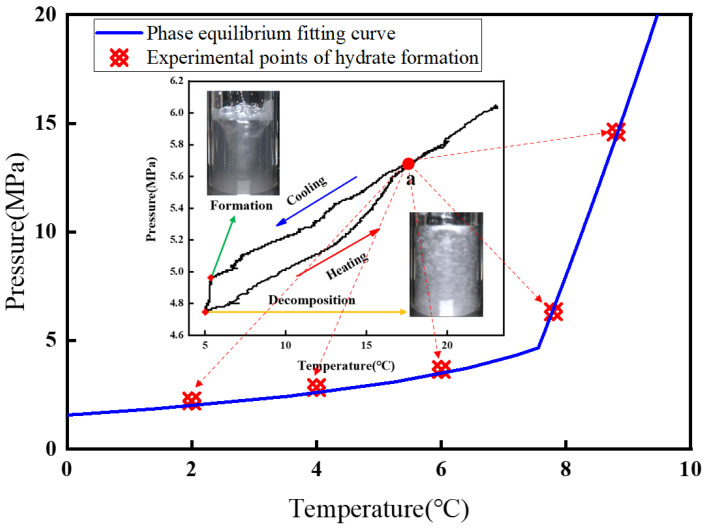
Experimental results and simulated curves of hydrate formation.

A constant-volume pressure method was used for the hydrate formation experiments, whereas an imaging method was employed for the hydrate phase-equilibrium experiments. In the hydrate formation experiments, the prepared solution was first loaded into the reactor, gas was then introduced until the preset pressure was reached, and after the temperature and pressure became stable, magnetic stirring was initiated at a speed of 200 rpm. The experiment was considered complete when, during the final 5 min, the rates of change in pressure and temperature were both less than 1%. In the hydrate phase-equilibrium experiments, the diesel-based oil–water system described in Section 2.2 was used, with a gas-to-oil ratio of 4:3 and an oil-to-water ratio of 1:1. Hydrates were formed by cooling and then dissociated by heating to obtain the complete formation and dissociation curves. The intersection point a of these two curves was taken to represent the actual condition under which hydrates exist, as shown in Figure 3.

### 2.4. Calculation Method for Hydrate Dynamic Parameters

(1) Gas Consumption

The amount and rate of gas consumption during hydrate formation are important parameters in the hydrate formation process. The gas consumption during the hydrate formation process is given by Equation (1):

(1) $$n_{g}=n_{0}-n_{t}=\frac{1}{R}(\frac{P_{0}V_{0}}{Z_{0}T_{0}}-\frac{P_{t}V_{t}}{Z_{t}T_{t}})$$

In the equation, *P* and *T* represent the pressure and temperature inside the reactor, and *V* is the gas volume in the reactor. *R* is the universal gas constant with a value of 8.314 J/(mol∙K). The compressibility factor is calculated using the Peng–Robinson equation of state [27]. The subscripts 0 and *t* represent the time points at *t* = 0 and *t*, respectively.

(2) Instantaneous Gas Consumption Rate

The instantaneous gas consumption rate characterizes the rate of hydrate formation at a given moment. The specific calculation formula is as follows:

(2) $$r=\frac{n_{i}-n_{i-1}}{t_{i}-t_{i-1}}$$

Here, *r* represents the hydrate gas consumption rate in mol∙min^−1^; and *n_i_* and *n_i_*_−1_ represent the gas consumption at times *t_i_* and *t_i_*_−1_ during hydrate formation in mol.

(3) Hydrate volume fraction

The hydrate volume reflects the compactness of hydrate cages and the stability of the crystal structure, and it is calculated as follows:

(3) $$\varphi_{h}=\frac{\frac{n_{g}M_{g}+N_{h}n_{g}M_{w}}{\rho_{h}}}{V_{w,\mathrm{ini}}+\frac{n_{g}M_{g}+N_{h}n_{g}M_{w}}{\rho_{h}}-\frac{N_{h}n_{g}M_{w}}{\rho_{w}}}$$

In the equation, *φ*_h_ denotes the hydrate volume fraction; *N*_h_ denotes the hydrate number, *N*_h_ = 5.75; *M*_g_ and *M*_w_ are the molar mass of gas and the molar mass of water, respectively, in kg/mol; and *ρ*_h_ and *ρ*_w_ are the density of hydrate and the density of water, respectively, with reference to the calculations by Qian et al. [28]. *V*_w,ini_ is the volume of the aqueous phase before hydrate formation in m^3^.

## 3. Results and Discussion

### 3.1. Effects of Temperature and Pressure on Hydrate Growth Behavior

Based on the composition determined in Section 2.2 and the hydrate formation curves shown in Figure 3, experiments were designed to investigate the effects of temperature and pressure on hydrate growth behavior, and the specific operating conditions are listed in Table 2. Different system pressures lead to changes in the gas phase state and therefore result in different hydrate formation morphologies. The experiments in this section cover three phase-state conditions, namely the gaseous state, the gas–liquid mixed state, and the liquid state, in order to explore the hydrate growth characteristics under different phase states induced by changes in temperature and pressure.

**Figure 4:**
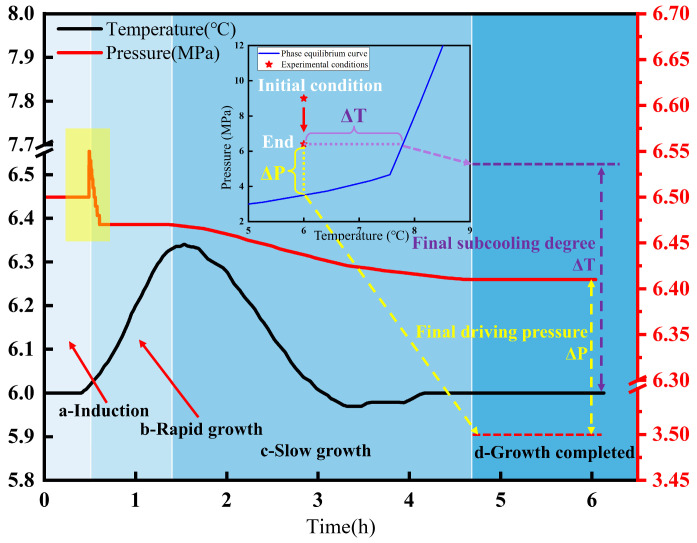
Pressure–temperature curve of the hydrate formation process at 6.5 MPa-6°C.

Taking Experiment A2 as an example, the gas composition phase diagram indicates that at 6.5 MPa the system was in a gas–liquid coexistence state, and the corresponding hydrate formation process is shown in Figure 4. Unlike the conventional hydrate formation curve, a pronounced pressure rise was observed during the rapid hydrate formation stage. According to the evolution characteristics of pressure and temperature, the entire hydrate formation process can be divided into four stages: the induction stage, the rapid formation stage, the slow formation stage, and the growth completion stage. At the end of hydrate growth, the experimental pressure–temperature state still deviates from the hydrate phase-equilibrium curve shown in Figure 3. In this study, the thermodynamic parameters used to characterize this deviation were defined on the basis of the phase-equilibrium relationship. For any experimental state (P1, T1), the equilibrium pressure corresponding to T1 is denoted as P0, and the driving pressure is defined as ΔP = P1 − P0. Likewise, the equilibrium temperature corresponding to P1 is denoted as T0 and the subcooling degree is defined as ΔT = T0 − T1. Accordingly, the values of ΔP and ΔT at the end of hydrate growth are referred to as the final driving pressure and final subcooling degree, respectively. As illustrated in Figure 4, the purple dashed line represents the equilibrium temperature obtained from the phase-equilibrium curve at the corresponding pressure, whereas the red dashed line represents the equilibrium pressure at the corresponding temperature.

**Figure 5:**
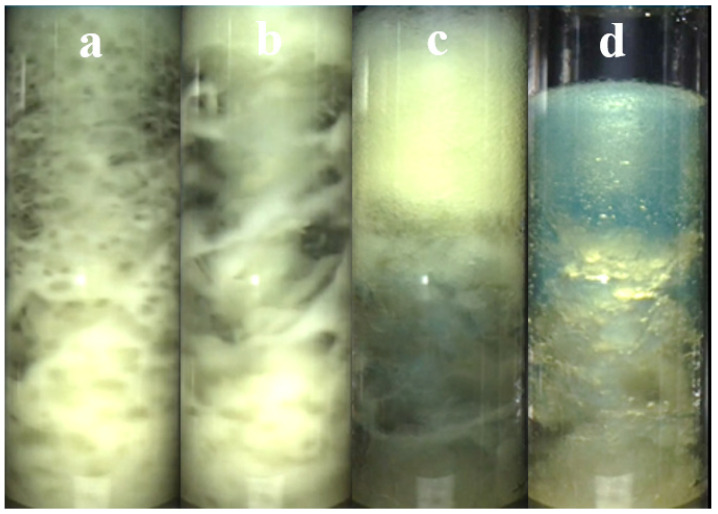
Morphology evolution in hydrate formation process: (**a**) No hydrate formation; (**b**) Rapid hydrate formation; (**c**) Hydrate deposition; (**d**) Slow hydrate growth with gas release.

At 6.5 MPa, the gas existed in both gaseous and liquid forms, and the liquefied gas dissolved into the oil phase, increasing the liquid-phase volume of the system and forming a uniform mixed phase under stirring (Figure 5a). Once hydrates formed, they rapidly settled to the bottom (Figure 5b). Hydrate agglomeration weakened the disturbance generated by the magnetic stirrer, leading to phase separation of the mixed solution (Figure 5c). At the same time, the dissolved gas in the oil phase escapes, accompanied by a phase transition (Figure 5d), which caused a temporary pressure spike. At this stage, hydrates formed in large quantities, the system temperature rose, and the process then entered the slow growth stage. A comparison between A2 and A3 showed that when the subcooling was reduced by 1 °C, the gas consumption decreased by 45%, the induction time increased ninefold, and the growth time was prolonged by 17.3%, indicating that less hydrate was formed and that the growth rate became slower. The hydrate growth behavior is consistent with the commonly reported trend, in which high subcooling and high driving pressure promoted hydrate nucleation and growth [29].

**Figure 6:**
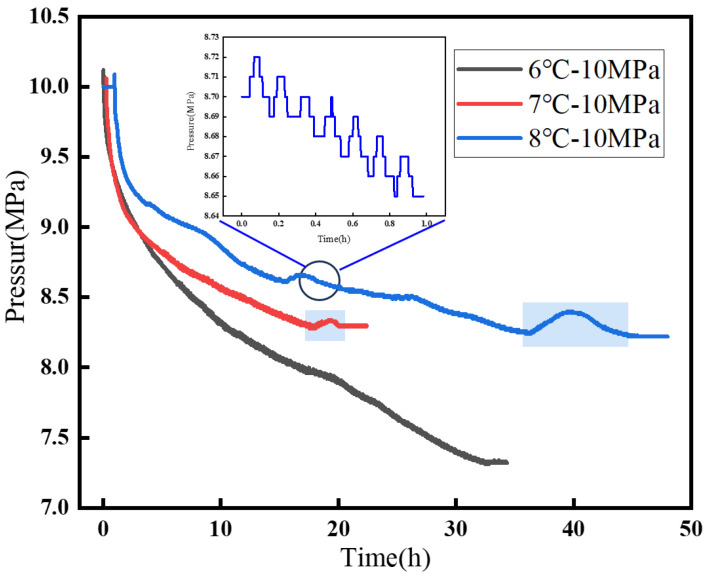
Hydrate formation process curve at 10 MPa.

At 10 MPa and higher pressures, the gas in the system became fully liquefied. Owing to the increase in pressure, the induction time became shorter, the hydrate growth rate increased, the growth time increased substantially, and the hydrate volume became larger. Although a pressure rise still occurred during hydrate formation, the pressure curve showed a fluctuating decline rather than the stepwise decrease observed at lower pressures (Figure 6). It was inferred that under high-pressure conditions, the hydrate growth rate was accelerated, leading to a more pronounced pressure drop. At the same time, as the pressure changed, dissolved gas continuously evolved from the liquid phase, which caused the pressure to rise. As a result, the curve exhibited a fluctuating stepwise decline rather than a horizontal stepwise decrease. In experiments performed at different subcooling levels, it was further observed that a period of pressure increase appeared at the final stage of hydrate formation (Figure 6). This behavior was considered to be related to the growth rate discussed above. Under low-subcooling conditions, the driving pressure at the final stage of hydrate formation was lower, and the growth rate was correspondingly the lowest [30]. When the gas consumption rate became lower than the gas evolution rate, that was, when the amount of gas consumed was smaller than the amount released, the pressure rose slowly. At 8 °C, the pressure fluctuation was more pronounced because the gas consumption rate was relatively low. After the system pressure gradually stabilized and the release of dissolved gas became steady, the amount of evolved gas decreased. Hydrate formation then continued, and the pressure decreased until hydrate formation was completed [31].

**Figure 7:**
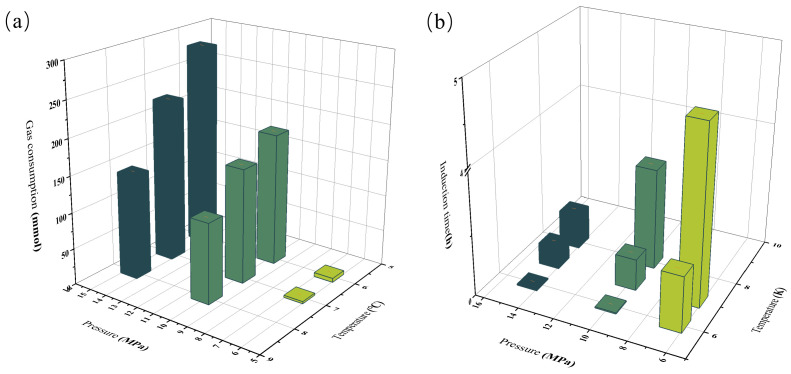
Hydrate gas consumption (**a**) and induction time (**b**) under various conditions.

Experiments A2–A10 were all conducted in systems containing liquid-phase gas, and the effects of temperature and pressure on hydrate growth behavior were similar to those described above. Figure 7 presents the gas consumption and induction time of the experiments, showing that as the driving pressure and subcooling increased, the amount of hydrate formed generally increased, whereas the hydrate formation time became shorter [32]. After the liquid-phase gas became mutually soluble with the oil phase in the system, a pressure rise occurred once hydrate formation began, during which dissolved gas evolution and gas uptake by hydrate cages took place simultaneously. Owing to the high heat-transfer capability of the oil phase, hydrates formed rapidly; meanwhile, the oil phase and liquid-phase gas further hinder the gas mass-transfer rate, which markedly prolongs the hydrate growth time [33]. In addition, the liquid-phase gas and the oil phase also affected the probability of hydrate nucleation and collisional agglomeration, leading to a substantial increase in induction time. Overall, the hydrates appeared as porous solids with multiple cavities and complex structures, and the presence of liquid-phase gas increased the space available for hydrate growth to some extent, thereby enlarging the volume of the hydrate plug [34].

**Figure 8:**
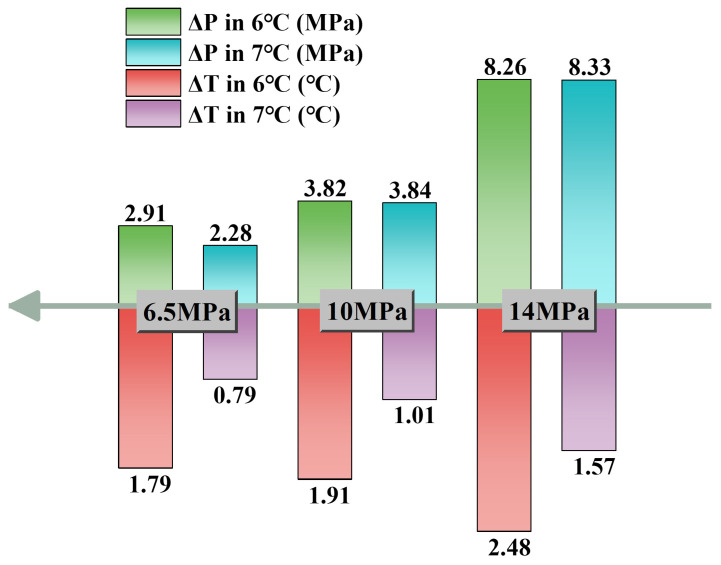
Final driving pressure and final subcooling degree at hydrate formation completion.

The magnitudes of subcooling and driving pressure reflect the strength of the driving force promoting hydrate formation. Table 2 lists the driving pressure and subcooling at the point when hydrate growth ceased, and Figure 8 shows the variations in terminal subcooling and terminal driving pressure at 6 °C and 7 °C. At the same temperature, the terminal driving pressure for hydrate formation increased with increasing system pressure. When the pressure increased from 6.5 MPa to 10 MPa, the terminal driving pressure for hydrate formation increased by about 31.2%. However, when the pressure further increased from 10 MPa to 14 MPa, the increase in terminal driving pressure exceeded the increase in system pressure, indicating that increasing pressure did not lead to a substantial rise in hydrate formation amount. Instead, the hydrate formation amount was controlled by other factors, such as the gas mass transfer rate. Compared with the effect of driving pressure on hydrate formation under the same operating conditions, the effect of subcooling was less significant, suggesting that under the present isothermal conditions, hydrate growth termination was more sensitive to the pressure-related driving force than to subcooling alone. Within the investigated range, the influence of subcooling was similar to that of driving pressure, and its overall trend also increased with increasing pressure.

The present results suggest that the gas phase state does not merely influence hydrate formation intensity, but fundamentally reorganizes the hydrate formation mechanism in the oil–water system. When the system remains in the gaseous state, hydrate nucleation is mainly controlled by gas–liquid interfacial contact and thermodynamic driving force, and the subsequent growth follows a relatively direct interfacial growth pathway. In contrast, once the gas enters a gas–liquid coexistence state or a fully liquid state, the hydrate formation process is no longer governed by a simple gas–water interfacial supply mode. Instead, gas dissolution into the oil phase, re-evolution from the liquid phase, redistribution of interfacial area, and local mass-transfer resistance jointly determine the nucleation probability, pressure response, and hydrate growth pathway. Therefore, the phase-state transition changes hydrate formation from a relatively interface-controlled crystallization process to a multiphase-coupled growth process involving dissolution-release competition and hindered transport.

### 3.2. Effect of CO_2_ Content on Hydrate Growth Behavior

CO_2_ concentration is an important factor affecting hydrate formation in wellbores. Based on the CO_2_ content in actual field-produced gas, three CO_2_ concentrations, namely 20%, 50%, and 80%, were selected for the experiments. The detailed experimental conditions and parameter results are listed in Table 3, with an experimental pressure of 10 MPa and a temperature of 8 °C. Using the gas composition in Table 1 as the baseline, the CO_2_ content in the system was increased by calculating the required amount of CO_2_ in the target system according to Dalton’s law of partial pressures, followed by adjustment using an additional CO_2_ cylinder. Different CO_2_ concentrations correspond to different phase diagrams. According to the phase diagrams, the gas remains in the gaseous state in the experiments with CO_2_ concentrations of 20% and 50%, whereas at a CO_2_ concentration of 80%, the gas became fully liquefied.

**Figure 9:**
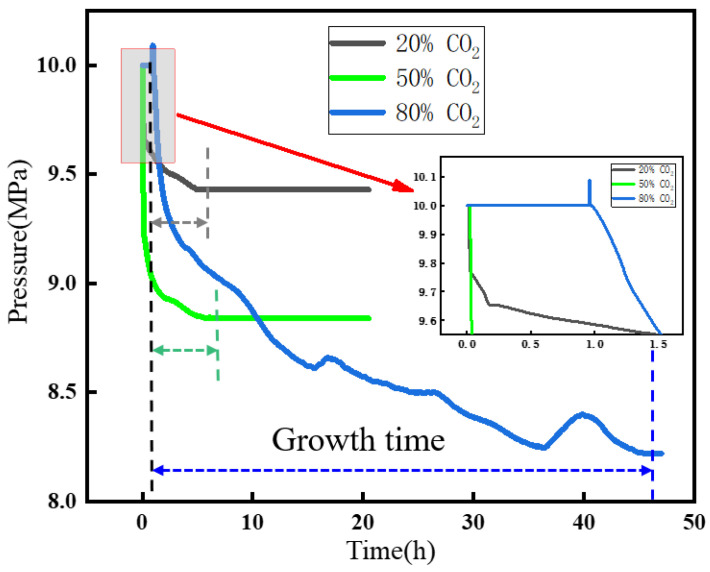
Pressure variation curves of gases with different CO_2_ contents.

Figure 9 shows the pressure evolution during hydrate growth at different CO_2_ concentrations. In systems B1 and B2, the gas remained in the gaseous state, and the induction time was much shorter than that in B3. The growth time was about 5–6 h, representing an 86% reduction in the overall growth time. This result indicates that the liquid phase significantly retards hydrate nucleation, because the higher molecular packing density after liquefaction reduces the probability of gas molecules entering hydrate cages. On the other hand, under gaseous conditions, the system with 50% CO_2_ exhibited a higher hydrate growth rate than that with 20% CO_2_, and its gas consumption was 98.4% higher than that of B1. For case B3, in which the gas was liquefied, the mass-transfer process during hydrate growth was clearly hindered. The growth curves of B1 and B2 were smoother, whereas the growth curve of B3 showed multiple fluctuations. These fluctuations involved both the vaporization of liquid-phase gas and gas consumption by hydrate formation, indicating that the pressure remained in a dynamic state throughout the process.

**Figure 10:**
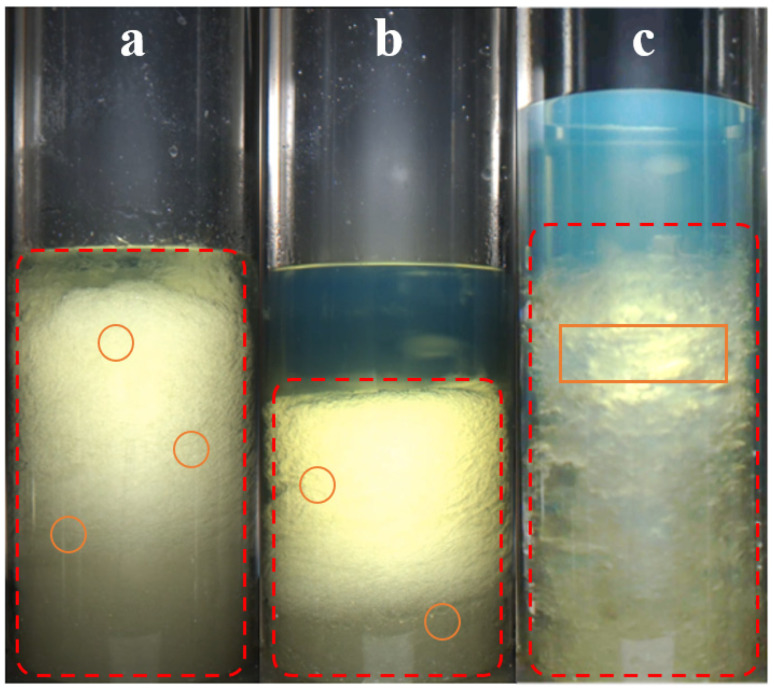
Hydrate morphologies of gases with different CO_2_ contents: (**a**) 20%; (**b**) 50%; (**c**) 80%.

Figure 10 shows the final hydrate morphologies at different CO_2_ concentrations. At a low CO_2_ concentration (20%), the hydrate particles were small and densely aggregated in a snowflake-like form. During formation, hydrates continuously developed at the gas–liquid interface, allowing sufficient growth. The overall hydrate mass exhibited relatively large pores and a foam-like morphology. At a CO_2_ concentration of 50%, hydrates rapidly accumulated at the top during formation and formed a dense hydrate layer, which then gradually sinks. As the gas–liquid contact area decreased, the subsequent growth depended mainly on dissolved gas. The hydrate morphology was more slush-like, and the hydrate lattice structure was stronger and more compact. Owing to the barrier effect of the oil layer, the lower hydrate mass was more loosely aggregated. When the CO_2_ concentration reaches 80%, the gas liquefied and becomes mutually soluble with the oil phase. Because the liquid-phase gas enlarged the mixed-phase space, hydrate formation differed from the conventional gas–liquid mass-transfer mode. Inside the mixed-phase region, hydrates nucleated and then partially aggregated into hydrate clusters, which gradually settled to the bottom. Because gas-phase mass transfer was absent, hydrate growth did not proceed through one-directional agglomerative growth, but instead expanded outward from the clusters in all directions. The liquid-phase gas molecules were larger, and the available growth space was also increased. As a result, the hydrate formation volume increased markedly, and the hydrates grew fully into an ice-like morphology with a relatively loose structure [35].

**Figure 11:**
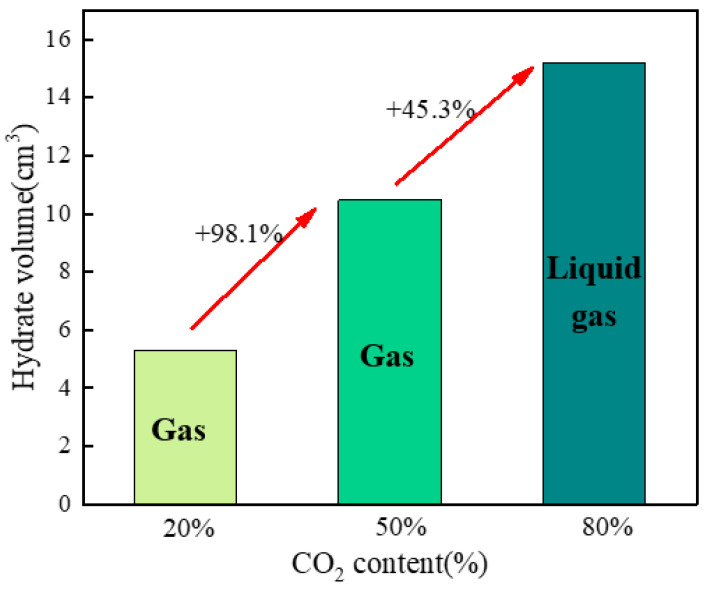
Hydrate volume at different CO_2_ contents.

When the CO_2_ concentration was 20%, the amount of hydrate formed in the system was relatively small, and the calculated hydrate volume was about 5.29 cm^3^. At this stage, the CH_4_ content in the system was relatively high, whereas the driving force for CH_4_ hydrate formation was comparatively weak, and its low solubility in water led to a slow gas-to-liquid mass-transfer rate, thereby limiting the amount of hydrate formed. Accordingly, the experimental observations showed that the hydrate structure was relatively loose and that the overall accumulated volume was small. When the CO_2_ concentration increased to 50%, the gas consumption of the system rose significantly to 76.8 mmol, and the hydrate volume reached about 10.48 cm^3^, which was approximately 1.98 times that of B1. This was because CO_2_ molecules provided a stronger driving force for hydrate formation, and their solubility in water was significantly higher than that of CH_4_, which accelerated gas diffusion into the aqueous phase and promoted the nucleation and growth of hydrate crystals. As a result, the hydrate formation rate in the system increased markedly, and the amount of hydrate formed rose substantially. When the CO_2_ concentration was further increased to 80%, the gas consumption reached 111.34 mmol, and the hydrate volume was about 15.20 cm^3^; compared with B1, the hydrate volume increased by about 2.87 times (Figure 11). The higher CO_2_ concentration further enhanced the driving force for hydrate formation in the system, enabling hydrate crystals to form rapidly and accumulate extensively in the reactor, which was experimentally manifested as a relatively dense solid-layer structure [36].

Overall, under the same temperature and pressure conditions, as the CO_2_ content in the mixed gas increases, the gas consumption of the system rose markedly, and both the mass and volume of the formed hydrates showed a significant upward trend. This indicates that CO_2_ plays an important promoting role in the formation of mixed-gas hydrates, and its higher solubility and stronger formation driving force can significantly enhance hydrate formation efficiency. To quantitatively characterize the differences in hydrate growth intensity under different phase-transition conditions, a phase-state factor was introduced, and a semi-empirical correlation model for hydrate growth rate was established by combining terminal subcooling and terminal driving pressure. According to the phase-state characteristics of the system, the phase-state factor was defined as 0 for the gaseous state, 0.5 for the gas–liquid coexistence state, and 1 for the liquid state.

(4) $$r=a+b\Phi +c\Delta T_{end}+d\Delta P_{end}$$

After further standardizing the variables based on the experimental results, the standardized regression equation under the influence of temperature, pressure, and CO_2_ content was obtained as follows:

(5) $$r^{*}=0.315\Phi^{*}-0.011\Delta T_{end}^{*}+0.412\Delta P_{end}^{*}(\text{P-T})$$

(6) $$r^{*}=-0.48\Phi^{*}+0.79\Delta T_{end}^{*}-0.09\Delta P_{end}^{*}({\mathrm{CO}}_{2})$$

Under P-T conditions, the relative contributions of terminal driving pressure, phase-state factor, and terminal subcooling to hydrate growth intensity are approximately 55.8%, 42.7%, and 1.5%, respectively. This result indicates that hydrate growth intensity is governed primarily by terminal driving pressure, followed by phase-state variation, whereas the overall contribution of terminal subcooling was relatively small. Similarly, under CO_2_ conditions, terminal subcooling had the most significant effect on the integrated growth index, followed by the phase-state factor, while the independent contribution of terminal driving pressure was relatively small. Based on the absolute values of the standardized regression coefficients, the relative contribution rates of the three factors were approximately 58.2% for terminal subcooling, 35.3% for the phase-state factor, and 6.5% for terminal driving pressure. These results suggested that as the CO_2_ content gradually increased, the tendency of the system toward liquefaction became stronger. However, if terminal subcooling and terminal driving pressure decreased substantially at the same time, liquid-state conditions did not necessarily correspond to a higher integrated growth intensity. In other words, the effect of CO_2_-content-induced phase transition on hydrate growth depended not only on whether liquefaction occurs, but also on whether the system driving force was maintained during the liquefaction process [37].

### 3.3. Effect of Hydrate Inhibitors

To prevent hydrate blockage and ensure flow safety, the injection of thermodynamic inhibitors is a commonly used method. Based on the preceding investigation of hydrate formation boundaries and formation behavior, the operating conditions for inhibitor injection experiments were determined, with methanol and MEG selected as the commonly used industrial inhibitors and the produced gas selected as the experimental gas; the detailed experimental conditions and resulting parameters are listed in Table 4.

**Figure 12:**
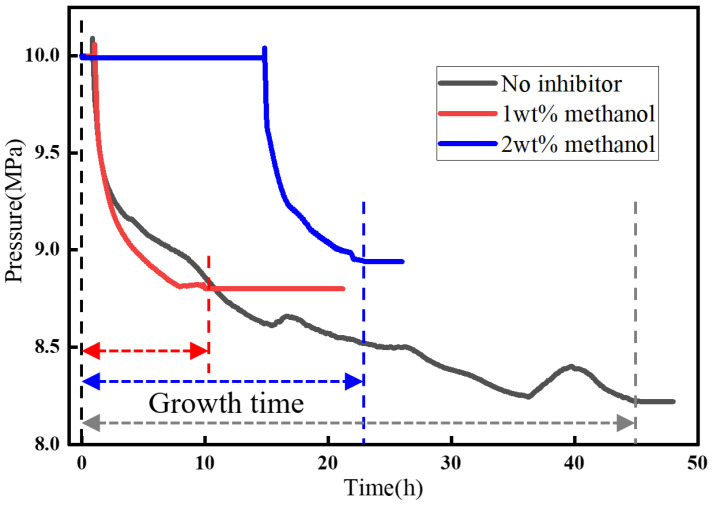
Hydrate formation curves under the influence of methanol.

Figure 12 showed the pressure variation during hydrate formation at 10 MPa and 8 °C after methanol was added. After the addition of 1 wt% methanol, based on the mass of the aqueous phase, the induction time increased by 4.6 times, the gas consumption decreased by 28.05%, and the growth time was reduced by 84.55%, indicating a clear inhibition effect. When the methanol concentration was increased to 2 wt%, the induction time became 44 times longer, the gas consumption was further reduced by 36.9%, the growth time was 7.45 h, and the average gas consumption rate decreased, indicating slow hydrate growth. When the methanol concentration was further increased to 3 wt%, no obvious sign of hydrate formation was observed within 48 h.

**Figure 13:**
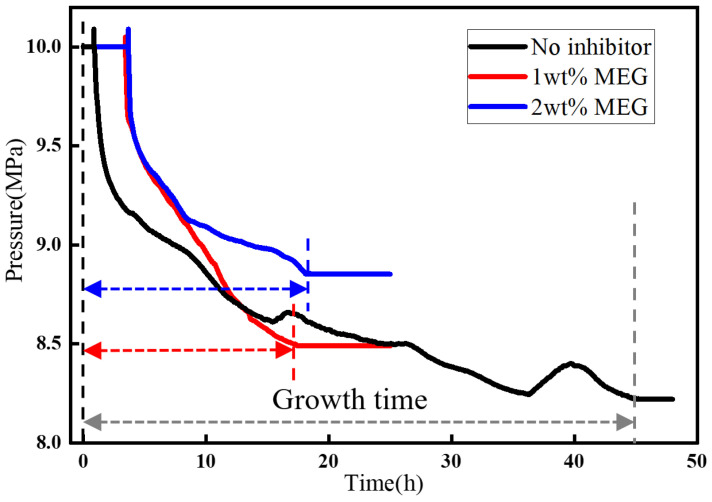
Hydrate formation curves under the influence of MEG.

**Figure 14:**
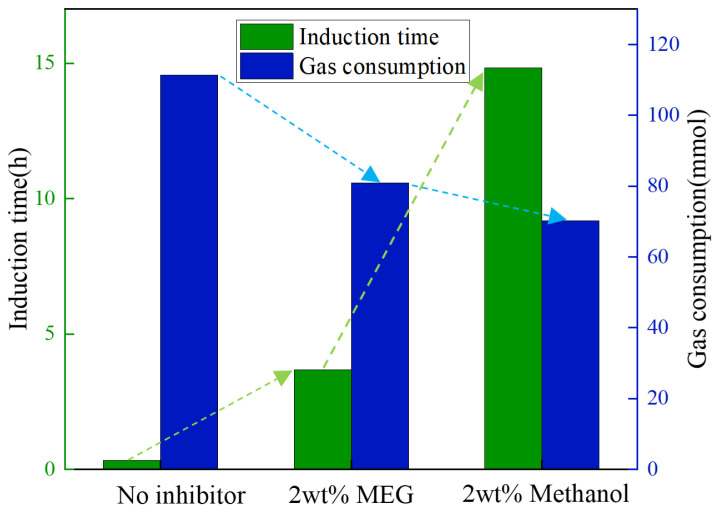
Inhibition effects of different inhibitors.

Figure 13 shows the pressure variation during hydrate formation after the addition of MEG. After the addition of 1 wt% MEG, based on the mass of the aqueous phase, the induction time was prolonged by a factor of 9.3, which was better than that achieved by methanol at the same mass fraction. However, the gas consumption decreased by only 9.1%, indicating that the inhibition effect was mainly reflected in the extension of induction time, while the overall amount of hydrate formed was not significantly suppressed. When the concentration was increased to 2 wt%, the induction time increased by only 7.9%, the gas consumption was further reduced by 27.3%, and no significant change was observed in the growth time. The average gas consumption rate decreased, and hydrate growth became slower. When the MEG concentration was further increased to 3 wt%, no obvious sign of hydrate formation was observed within 48 h. Since no hydrate formation was observed at an inhibitor concentration of 3 wt% for either inhibitor, Figure 14 compares the inhibition performance of the two inhibitors at 10 MPa and 8 °C. At the same concentration, the induction time with methanol was 3.04 times longer than that with MEG, and the gas consumption was further reduced by 13.22%, indicating that methanol exhibited better inhibition performance than MEG.

**Figure 15:**
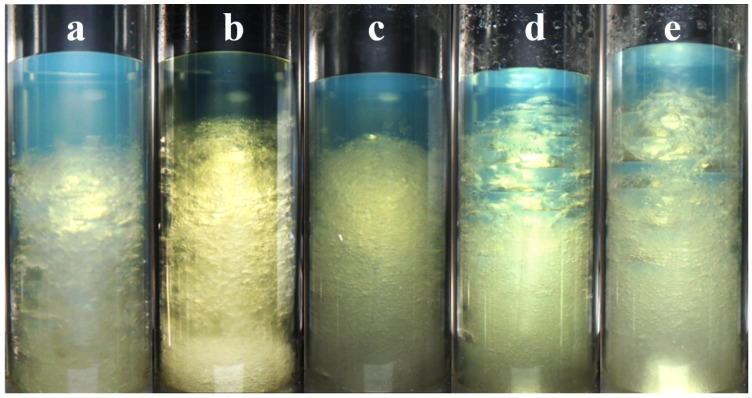
Hydrate formation states: (**a**) without inhibitor; (**b**) 1 wt% methanol; (**c**) 2 wt% methanol; (**d**) 1 wt% MEG; (**e**) 2 wt% MEG.

Figure 15 shows the hydrate formation states after the addition of inhibitors. In the methanol-added group, the hydrate formation state was similar to that observed without inhibitor addition. In contrast, the hydrate volume increased markedly in the MEG-added group. It is inferred that the presence of diesel and MEG may alter the hydrogen-bond strength between water molecules [38] and reduce the affinity between hydrate cages. As a result, hydrate formation may proceed more freely or develop a looser structure, which is macroscopically manifested as larger crystal structures and, consequently, a greater overall hydrate volume [39]. In addition, in the high-concentration inhibitor groups, the hydrates are mostly in a flowable slush-like form, and no solid plug formed at the bottom.

**Figure 16:**
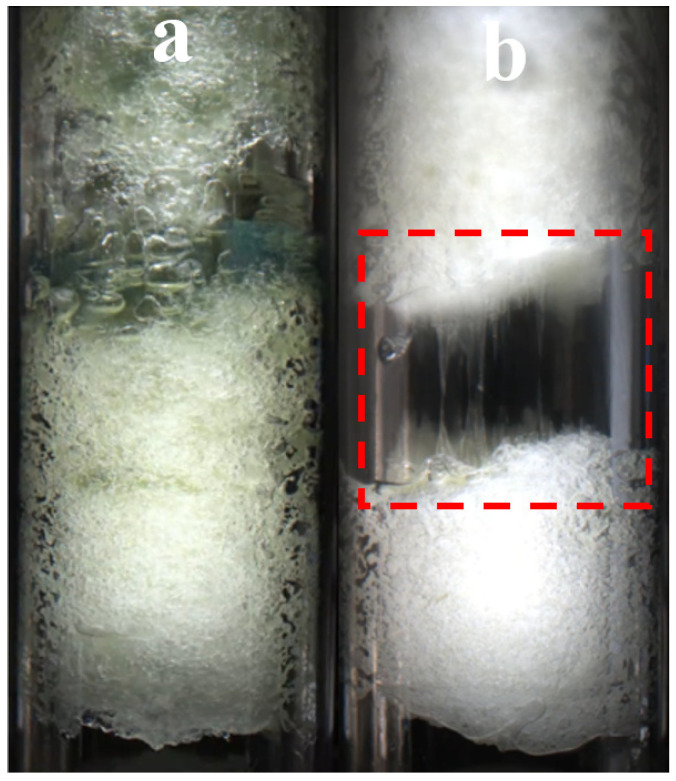
Hydrate morphology during dissociation under conditions C1 (**a**) and C2 (**b**).

Figure 16 shows the morphological changes during hydrate decomposition under conditions C1 and C2. Unlike the conventional fracture mode during dissociation shown in panel (a), the experimental group containing 1 wt% methanol exhibited a pronounced filament-drawing behavior during dissociation rather than direct fracture (b). This phenomenon is attributed to the effect of methanol, as a polar solvent, on the hydrogen bonding formed during hydrate formation, which makes the crystal structure of the hydrate relatively softer and enhances its ductility [40]. In addition, the pressure change during depressurization-induced dissociation lowered the local temperature within the system, which may lead to hydrate formation even during the dissociation process. On the other hand, the polarity of methanol may cause non-uniform expansion or contraction of the hydrate during dissociation, making gas more likely to accumulate at fracture points of the hydrate. As a result, the hydrate layer fractures while filamentous connections remain between the broken parts [41]. From a mechanistic perspective, the filament-drawing phenomenon implies that the hydrate structure in the methanol-containing system may experience non-uniform local failure during depressurization. Rather than collapsing through abrupt fragmentation, some regions remain temporarily connected and stretch into filament-like bridges before final rupture. This suggests that methanol may modify the local bonding environment, gas-release pattern, and structural stress redistribution during hydrate dissociation, thereby affecting both the macroscopic dissociation morphology and the underlying fracture behavior.

**Figure 17:**
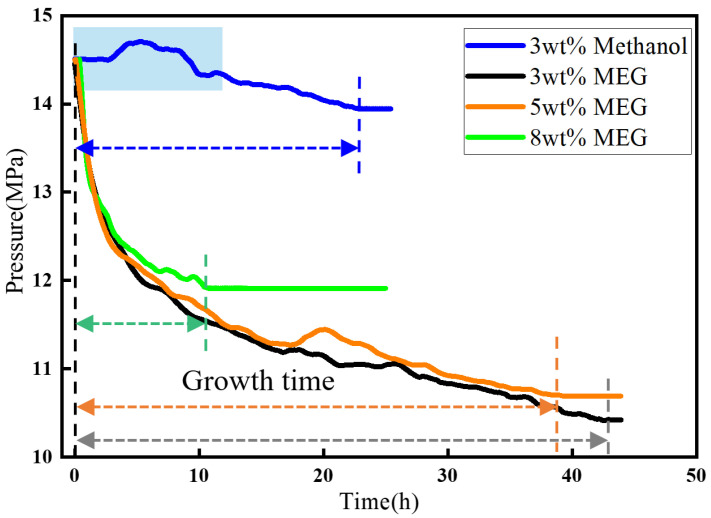
Hydrate growth pressure curve with inhibitor injection at 14.5 MPa.

Figure 17 presents the pressure behavior during hydrate formation after the injection of methanol and MEG inhibitors at 14.5 MPa and 8 °C, where the concentrations of both inhibitors were gradually increased under these temperature and pressure conditions. Based on the experimental results, the addition of 3 wt% MEG did not reduce hydrate formation, but instead led to a greater amount of hydrate being formed. This may be attributed to the fact that MEG increased the fluidity of the system during hydrate formation, while the degree of agglomeration of the hydrate shell in the initial formation stage affects both the formation rate and the growth time. When the MEG concentration was further increased to 5 wt%, an inhibitory effect appeared, with gas consumption decreasing by 6.14% and the growth time increasing by a factor of 5.79. When the MEG concentration was further raised to 8 wt%, the inhibition effect became significant, with the induction time increasing by 68.96%, the gas consumption decreasing by 31.11%, and the growth time increasing by 56.21%. After depressurization, the hydrates dissociated and fractured, and the total amount of hydrate formed was relatively small. When the MEG concentration was further increased to 10 wt%, no obvious hydrate formation was observed within 24 h. After the addition of 3 wt% methanol, a clear inhibition effect was immediately observed, as the peak value during the rapid hydrate formation stage decreased (Figure 17) and the hydrate growth rate was markedly reduced. As a result, the period during which the pressure remained above the initial pressure became longer, and the overall gas consumption decreased by 92.29%. When the methanol concentration was further increased to 5 wt%, no obvious hydrate formation was observed within 24 h.

Overall, these results demonstrate that gas phase transition fundamentally governs hydrate formation behavior in the CO_2_-rich oil–water system. The hydrocarbon phase plays an essential role. In particular, the hydrocarbon phase affects hydrate formation through phase partitioning and dissolution behavior after gas liquefaction, interfacial redistribution and growth pathway variation, as well as heat/mass transfer resistance and agglomeration behavior. Consequently, hydrate nucleation, pressure response, growth pathway, and final morphology are all significantly altered. Such behavior is highly relevant to real deepwater field conditions, where CO_2_-rich associated gas, oil, and water coexist under high-pressure and low-temperature environments, particularly during shut-in, restart, and inhibitor injection operations. Therefore, the present study provides a more realistic basis for understanding hydrate risk in actual multiphase production and transportation systems.

## 4. Conclusions

Based on a fully visualized high-pressure experimental apparatus, this study systematically investigated the hydrate formation characteristics of CO_2_-rich mixed gas in oil–water systems under different phase states, as well as the mechanisms of inhibitor action, and the main conclusions are as follows:

(1) Variations in temperature and pressure can significantly alter the gas phase state of the system and further affect hydrate nucleation and growth behavior. As the system transitions from the gaseous state to the gas–liquid coexistence state and then to the liquid state, hydrate formation is accompanied by a sudden pressure rise and a reduced mass-transfer rate. Overall, high driving pressure and high subcooling are favorable for shortening the induction time, increasing gas consumption, and promoting hydrate growth.
(2) After the liquid-phase gas became mutually soluble with the oil phase, the heat-transfer and mass-transfer processes, as well as the nucleation and agglomeration modes of the system, are altered. The liquid-phase gas enlarged the space available for hydrate growth, causing the formation process to involve a dynamic competition between dissolved-gas evolution and gas consumption by hydrate formation. As a result, the pressure curve showed a fluctuating decline, and local pressure recovery appeared at the final stage of formation.
(3) An increase in CO_2_ concentration generally promoted hydrate formation, and both the amount and volume of hydrates increased markedly. Once the system enters the liquid state, the mass-transfer process became hindered, resulting in a significant extension of induction time, a lower growth rate, and a longer growth time.
(4) Both methanol and MEG could effectively inhibit hydrate formation in oil–water systems, but methanol showed overall better inhibition performance than MEG, as it could significantly prolong the induction time and reduce gas consumption and growth rate even at lower concentrations. Moreover, methanol alters the dissociation morphology and fracture behavior during hydrate dissociation, indicating that it exerted a stronger influence on the hydrate crystal structure and hydrogen-bonding interactions.

## Figures and Tables

**Table 1:** Composition of the gas used for hydrate experiments.

Component	mol%	Component	mol%	Component	mol%
N_2_	1.580	C1	10.273	C3	2.371
CO_2_	80.654	C2	1.971	iC4	1.180
nC4	1.971				

**Table 2:** Experimental parameters and results of hydrate formation.

Case	Pressure (MPa)	Temperature (°C)	Induction Time (h)	Gas Consumption (mmol)	Growth Time (h)	Final Subcooling Degree (°C)	Final Driving Pressure (MPa)	Gas State
A1	3.5	2	0.02	69.3	4.11	1.09	0.14	Gas
A2	6.5	6	0.46	56.6	3.64	1.79	2.91	G–L
A3	6.5	7	4.66	31.1	4.27	0.79	2.28	G–L
A4	6.5	8	0	0	0	0	0	G–L
A5	10	6	0.01	183.62	31.43	1.91	3.82	Liquid
A6	10	7	0.26	158.53	32.15	1.01	3.84	Liquid
A7	10	8	0.33	111.34	44.02	0.12	0.23	Liquid
A8	14.5	6	0.01	220.44	11.30	2.48	8.26	Liquid
A9	14.5	7	0.18	141.63	10.49	1.57	8.33	Liquid
A10	14.5	8	0.29	277.58	6.12	0.42	3.34	Liquid

**Table 3:** Experimental Parameters and Results of hydrate Formation under Different CO_2_ Contents.

Case	CO_2_ Content	Induction Time (h)	Gas Consumption (mmol)	Growth Time (h)	Final Subcooling Degree (°C)	Final Driving Pressure (MPa)	Gas State
B1	20	0.03	38.7	5.08	10.1	7.42	Gas
B2	50	0.02	76.8	6.16	7.28	6.57	Gas
B3	80	0.33	111.34	44.02	0.12	0.23	Liquid

**Table 4:** Experimental parameters and results of hydrate inhibitor injection.

**Case**	**Pressure** **(MPa)**	**Temperature** **(°C)**	**Inhibitor**	**Inhibitor Content (wt%)**	**Induction Time (h)**	**Gas Consumption** **(mmol)**	**Growth Time (h)**
C1	10	8	/	0	0.33	111.34	44.02
C2	10	8	Methanol	1 wt%	1.85	80.12	6.8
C3	10	8	Methanol	2 wt%	14.83	70.20	7.45
C4	10	8	Methanol	3 wt%	0	0	0
C5	10	8	MEG	1 wt%	3.40	101.14	13.85
C6	10	8	MEG	2 wt%	3.67	80.9	14.22
C7	10	8	MEG	3 wt%	0	0	0
C8	14.5	8	/	0	0.29	277.58	6.12
C9	14.5	8	Methanol	3 wt%	3.17	21.4	21.83
C10	14.5	8	Methanol	5 wt%	0	0	0
C11	14.5	8	MEG	3 wt%	0.12	267.46	38.8
C12	14.5	8	MEG	5 wt%	0.13	260.63	41.6
C13	14.5	8	MEG	8 wt%	0.49	191.22	9.56
C14	14.5	8	MEG	10 wt%	0	0	0

## Data Availability

The original contributions presented in this study are included in the article. Further inquiries can be directed to the corresponding authors.

## Nomenclature

$n_{g}$	the amount of gas consumed in the process of hydrate formation (mol)
$P_{0}$	the pressure in the reactor at the beginning of hydrate formation (MPa)
$T_{0}$	the temperature in the reactor at the beginning of hydrate formation (K)
$V_{0}$	the volume of the gas phase in the reactor at the initial moment (mL)
$Z_{0}$	the compression factor at the initial moment
R	the universal gas constant (8.314 KJ/mol)
$P_{t}$	the pressure in the reactor at time t (MPa)
$T_{t}$	the temperature in the reactor at time t (K)
$V_{t}$	the volume of the gas phase in the reactor at time t (mL)
$Z_{t}$	the compression factor at time t
$n_{0}$	the amount of gas at the beginning (mol)
$n_{t}$	the amount of gas at the time t (mol)
r	gas consumption rate of hydrate formation (mol/h)
$\varphi_{h}$	the volume fraction of the hydrate
$N_{h}$	the hydrate number (5.75)
$M_{g}$	the molar mass of gas (kg/mol)
$M_{w}$	the molar mass of water (kg/mol)
$\rho_{h}$	the density of hydrate (kg/m^3^)
$\rho_{w}$	the density of water (kg/m^3^)
$V_{w,\mathrm{ini}}$	the volume of the aqueous phase before hydrate formation (m^3^)
P	the pressure at a certain moment (MPa)
T	the temperature at a certain moment (K)
